# LONG-TERM SURVIVAL AFTER LAPAROSCOPIC TOTAL GASTRECTOMY FOR EARLY AND ADVANCED GASTRIC CANCER. SINGLE CENTER EXPERIENCE IN 100 CASES

**DOI:** 10.1590/0102-6720202400050e1844

**Published:** 2024-12-16

**Authors:** Enrique NORERO, Marco CERONI, Cristian MARTINEZ, Rodrigo MUÑOZ, Ricardo MEJIA, Emilio MORALES, Ignacio OBAID, Paulina GONZALEZ

**Affiliations:** 1 Pontificia Universidad Católica de Chile, Hospital Dr. Sotero del Rio, Esophagogastric Surgery Unit, Digestive Surgery Department – Santiago, Metropolitan Region, Chile.

**Keywords:** Gastrointestinal Neoplasms, Laparoscopy, Gastrectomy, Postoperative Complications, Survival, Neoplasias Gastrointestinais, Laparoscopia, Gastrectomia, Complicações Pós-Operatórias, Sobrevida

## Abstract

**BACKGROUND:**

Laparoscopic gastrectomy offers advantages in the postoperative period compared to the open approach. Most studies have been performed on distal gastrectomies; however, laparoscopic total gastrectomy (LTG) is not universally accepted. AIM: The aim of this study was to assess the results of LTG, on postoperative morbidity outcomes and long-term survival.

**METHODS:**

This is a retrospective cohort study from a prospective database of patients who underwent LTG, from 2005 to 2022, due to early and advanced gastric cancer. A totally laparoscopic technique was utilized, and the Roux-en-Y reconstruction was performed in all cases. Postoperative complications and long-term survival were evaluated.

**RESULTS:**

A total of 100 patients were included (men 57, age 64 years, and body mass index 26). A D2 lymphadenectomy was performed in 68 cases. The postoperative hospitalization period was 8 days (6–62 days). Postoperative complications occurred in 26%, with 7% esophago-jejunal anastomosis leak, 4% abdominal collections, and 2% gastrointestinal bleeding. In 7% of cases, the complication was considered Clavien 3 or greater. Operative mortality was 1%. The pathology findings confirmed advanced gastric cancer in 50 cases. The median lymph node count was 38, and surgery was considered R0 in 99%. The median follow-up was 50 months. Overall 5-year survival was 74%. Survival in T1 cases was 95% at 5 years. For stage I, survival was 95%, and for stages II and III, it was 52% and 43%, at 5 years, respectively.

**CONCLUSIONS:**

These results support the feasibility and oncological adequacy of minimally invasive total gastrectomy. Postoperative morbidity has an acceptable rate. Long-term survival was in accordance with the disease stage.

## RESUMO

**RACIONAL:**

A gastrectomia laparoscópica oferece vantagens no pós-operatório em comparação à abordagem aberta. E a gastrectomia total laparoscópica não é universalmente aceita. OBJETIVOS: Avaliar os resultados da gastrectomia total laparoscópica, nos resultados de morbidade pós-operatória e sobrevida em longo prazo.

**MÉTODOS:**

Este é um estudo de coorte retrospectivo de um banco de dados prospectivo de pacientes submetidos à gastrectomia total laparoscópica, de 2005 a 2022, devido a câncer gástrico precoce e avançado. Foi utilizada a técnica totalmente laparoscópica e a reconstrução em Y de Roux foi realizada em todos os casos. Complicações pós-operatórias e sobrevida em longo prazo foram avaliadas.

**RESULTADOS:**

Foram incluídos 100 pacientes (homens 57, idade 64 anos, IMC 26). Linfadenectomia D2 foi realizada em 68 pacientes. O tempo de hospitalização pós-operatório foi de 8 dias (6-62 dias). Complicações pós-operatórias ocorreram em 26%, com 7% de fístulas da anastomose esôfago-jejunal. Em 7% dos casos a complicação foi considerada Clavien 3 ou superior. A mortalidade operatória foi de 1%. Os achados anátomo-patológicos confirmaram câncer gástrico avançado em 50 casos. A mediana da contagem de linfonodos foi de 38 e a cirurgia foi considerada R0 em 99%. O acompanhamento médio foi de 50 meses. A sobrevida global em 5 anos foi de 74%. A sobrevida nos casos T1 foi de 95% em cinco anos. Para o estágio I a sobrevida foi de 95% e para os estágios II e III foi de 52% e 43% em cinco anos, respectivamente.

**CONCLUSÕES:**

Os resultados apoiam a viabilidade e adequação oncológica da gastrectomia total minimamente invasiva. A morbidade pós-operatória tem uma taxa aceitável. A sobrevida em longo prazo esteve de acordo com o estágio da doença.

## INTRODUCTION

Since the first laparoscopic gastrectomy in gastric cancer (GC) was performed three decades ago^23^, there is a progressive interest in this technique. It is mainly because most laparoscopic abdominal procedures have proven to be associated with lower morbidity and faster patient recovery^40^.

A main concern over the past decades was the oncological equivalence and long-term survival with the laparoscopic approach. This has been studied in several publications, and currently, there are randomized controlled trials that demonstrate an equivalent oncological result with laparoscopic surgery for early^17,18,20,22,28^ and advanced GC^6,11,13,27,38,41,45^.

However, most of the experience with laparoscopic gastrectomy comes from distal gastrectomy^6,11,13,17,18,20,22,27,38,45^, mainly because in the Eastern countries with a high incidence of GC, like Japan, Korea, and China, the distally located GC is more prevalent than in Western countries. Thus, laparoscopic distal gastrectomy has been widely studied.

Laparoscopic total gastrectomy (LTG) is not universally accepted, and few studies have evaluated its results^12,16,25,28,42^. The major concerns about LTG are the requirement for more extensive lymph node dissection and the safety of the reconstructive phase of the surgery^3,4,9,24,43,46^.

In our center, a public hospital in a country with a high incidence of GC, we started performing LTG in 2006^32^. And now we have a mature experience with LTG and long-term follow-up.

The aims of the study were to assess the postoperative morbidity outcomes and the long-term survival after LTG.

## METHODS

### Patients

This is a retrospective cohort study, from a prospective database of patients who underwent laparoscopic gastrectomy. We included all consecutive cases of LTG.

We included patients who had noncurative endoscopic resection requiring an LTG^31^. We included patients with neoadjuvant chemotherapy and LTG that required a conversion to open surgery. We excluded patients with a laparoscopic subtotal gastrectomy, LTG performed by other histology different from adenocarcinoma, and laparoscopic totalizations.

### Preoperative workup

The preoperative evaluation included an upper gastrointestinal endoscopy, biopsies, complete blood count, liver function tests, electrocardiogram, and nutritional evaluation. Preoperative imaging included a thorax-abdomen-pelvis computed tomography (CT). All cases were discussed at a multidisciplinary meeting.

LTG was decided depending on tumor location. Lymph node dissection was performed according to the Japanese guidelines^1^. The decision between LTG and open total gastrectomy was based on the team and surgeon preference and experience. All surgeries were performed by attending surgeons with experience in open gastrectomy and a developing learning curve for LTG.

### Laparoscopic total gastrectomy surgical technique

Our LTG technique has been previously described^32^. Briefly, a pneumoperitoneum with CO_2_ at 15 mmHg was established, and six laparoscopic ports and a 30º scope were employed. The duodenum is divided using a 60-mm linear stapler, and it is reinforced with an invaginating 3.0 vicryl running suture. The esophagus was also divided using a 60-mm linear stapler, and the surgical specimen was extracted through a 6-cm suprapubic incision. A frozen section of the esophageal margin was evaluated in gastric upper third and esophagogastric junction cancers. An intracorporeal esophago-jejunal anastomosis was performed with a Roux-en-Y reconstruction. Different techniques of esophago-jejunal anastomosis were used, but in most cases, we performed an intracorporeal hand-sewn method, as previously described^33^. We used two drains, directed to the esophago-jejunal anastomosis and the duodenal stump.

### Postoperative period

In the postoperative period, immediate extubation was favored, and patients began physical and respiratory therapy as soon as possible. In the standard postoperative hospitalization, patients spent the first night in a monitored bed, and on the first postoperative day, they were transferred to the general surgical ward. Epidural analgesia and nasogastric tube were not utilized. An oral contrast study was performed with water-soluble contrast 3–4 days after surgery to rule out esophago-jejunal anastomosis fistula. The drains were extracted when the output was under 100 cc, and there were no signs of fistula. We performed a laboratory test on days 3, 5, and 7. The patients were discharged when they were able to tolerate a soft diet for 24 h. We used a standardized protocol for postoperative management.

### Complications

All deviations from a normal postoperative course of elective gastrectomy for up to 30 days or during the hospital stay were considered postoperative complications. Readmission was considered for up to 60 postoperative days.

The appearance of contrast outside the esophago-jejunal anastomosis in an oral contrast study or CT scan or by direct evaluation at reoperation was defined as a leak. The impossibility of advancing a standard diagnostic upper digestive endoscopy through the anastomosis or the need for endoscopic dilation was defined as esophago-jejunal anastomosis stenosis. Complications were evaluated according to the Clavien-Dindo classification^5^.

### Follow-up

Staging was based on the seventh edition of TNM-AJCC^30^ according to definitive pathology. The follow-up program consisted of a physical examination, laboratory blood tests, endoscopy, abdominal ultrasonography, and thorax-abdomen-pelvis CT scan up to 5 years after surgery.

Demographic data, bleeding, operating time, esophageal-jejunal anastomosis methods, postoperative complications, and long-term survival were evaluated. A specialized nurse, prospectively, collected the data.

### Statistical analyses

Statistical analyses were performed using SPSS version 22, Inc., Chicago, IL, and Minitab 15. Categorical variables are expressed in percentages (%), and quantitative values are expressed as the median (range). Survival curves were estimated according to the Kaplan-Meier method.

The local Ethics Committee approved the study, and the informed consent of patients was waived because of the retrospective nature of the study.

## RESULTS

We included 100 patients, 57 men, aged 64 (22–87) years, body mass index (BMI) 26 (16–40). Notably, 64 patients were either overweight or obese. Seventy-six patients had comorbidities, and 36 had a history of previous laparotomies (Table 1). Five patients had a previous noncurative endoscopic submucosal dissection, and three had preoperative chemotherapy.

**Table 1:** Patients treated with laparoscopic total gastrectomy. Demographics and surgery details.

	n=100
Sex	
Male	57
Female	43
Age	64 (22–87)
BMI	26 (16–40)
Comorbidities	
Arterial hypertension	31
Diabetes	17
Heart disease	9
Pulmonary disease	8
Chronic renal failure	4
Chronic liver disease	1
Stroke	1
ASA score	
I	24
II	64
III	12
Previous laparotomy	36
Previous supraumbilical laparotomy	20
Tumor location	
Esophagogastric junction	6
Upper third of stomach	45
Middle third of stomach	36
Lower third of stomach	10
Lymph node dissection	
D1	19
D1+	13
D2	68
Conversion to open surgery	6
Roux-en-Y reconstruction	
Antecolic	24
Retrocolic	73
Esophago-jejunal anastomosis method	
Hand-sewn method	90
Single-layer	8
Two-layer	82
Linear stapler overlap method	3
Circular stapler Orvil^TM^ method	2
Circular stapler*	5
Bleeding	150 cc (10–600)
Operative time	330 min (180–530)
Length of hospital stay	8 days (6–62)

BMI: body mass index; ASA: American Society of Anesthesiologists; D: lymph nodes dissection; cc: centiliters.

*Cases converted to open surgery.

Most of the tumors were located in the upper and middle third of the stomach, in 45 and 36 patients, respectively. In six patients, there was a focal invasion of the esophagogastric junction (Table 1).

A D2 or D1+ lymphadenectomy was performed in 68 and 13 cases, respectively (Table 1).

A conversion to open surgery was necessary for six patients, and in five of them, this was necessary for the resective part of the operation, due to bleeding, staple misfire, and difficult dissection. And in one case, a conversion was decided during the esophago-jejunal anastomosis phase. A Roux-en-Y reconstruction was performed in all cases. The access route for the alimentary limb was retrocolic in 73 patients.

A hand-sewn esophago-jejunal anastomosis was performed in 90 cases, and a mechanical suture was used in the remaining 10 cases. A linear stapler overlap method was employed in three cases, and in one of these cases, there was a submucosal stapler firing on the esophageal side of the anastomosis, requiring conversion to open surgery, lower mediastinal dissection, and reanastomosis with a circular stapler. In two cases, we used an Orvil^TM^ device. The remaining five cases were performed with a circular stapler in the cases of conversion (Table 1).

Bleeding was 150 cc (10–600), and operating time was 330 min (180–530). The postoperative length of stay was a median of 8 days (6–62).

Postoperative complications occurred in 26 surgeries. The most frequent complications were esophago-jejunal anastomosis fistula with 7%, intrabdominal abscess-collection 4%, chyle leak 2%, and gastrointestinal bleeding 2% (Table 2).

**Table 2:** Postoperative complications after laparoscopic total gastrectomy.

	n (%)
Intrabdominal	
Esophagojejunostomy fistula	7 (7)
Intrabdominal abscess/collection	4 (4)
Quile leak	2 (2)
Intraluminal hemorrhage	2 (2)
Biliary fistula	1 (1)
Pancreatic fistula	1 (1)
Wound hematoma	1 (1)
Hemobezoar	1 (1)
Intraperitoneal bleeding	1 (1%)
Medical	
Pulmonary embolism	2 (2)
Acute renal failure	2 (2)
Urinary tract infection	2 (2)
Pneumonia	1 (1)
Pseudomembranous colitis	1 (1)
Total complications	26 (26)
Clavien 3	5 (5)
Clavien 4	1 (1)
Clavien 5	1 (1)

In 7% of cases, the complication was considered Clavien 3 or greater because of a reoperation. These cases were represented by four cases of esophago-jejunal anastomosis fistula that required peritoneal washing and drainage, one abscess drainage, one case of peritoneal bleeding, and one case of hemobezoar located at the jejuno-jejunal anastomosis. Operative mortality was 1%, this was a patient who presented esophago-jejunal anastomosis fistula requiring a reoperation, in spite of reoperation and intensive care management, the patient died from persistent sepsis and multiple organ failure. None of the patients with medical complications had severe complications (Table 2).

Two patients had delayed complications associated with gastrectomy, and one case of esophago-jejunal anastomosis stenosis was successfully treated with endoscopic dilation. And one case of transverse colon stenosis required surgical resection, 3 months after LTG, due to mesocolic vessel injury that required a colonic resection.

The mechanical esophago-jejunal anastomosis methods were used in five patients, three linear staplers, and two Orvil^TM^. Two (40%) of these five patients presented anastomotic fistula. The hand-sewn esophago-jejunal anastomosis technique presented a 4% fistula rate.

The pathology findings confirmed early GC (T1) in 50 cases and advanced GC (T2–T4) in 50 cases. There were 19 cases with serosal invasion (Table 3). There was lymph node involvement in 33 patients. The median lymph node count was 38 nodes. Surgery was R0 in 99%, and there was one definitive positive margin case in the proximal margin at the esophagus. Four cases had stage IV disease, because of peritoneal disease in three cases, and liver metastases in one case and were resected for symptom palliation (Table 3).

**Table 3:** Pathologic staging and margin status.

T	
T1	50
T2	16
T3	16
T4A	19
N	
N0	67
N1	9
N2	11
N3	13
M	
M0	96
M1	4
Stage I	59
IA	48
IB	11
Stage II	17
IIA	4
IIB	13
Stage III	20
IIIA	12
IIIB	1
IIIC	7
IV	4
R	
R0	99
R1	1
R2	0

T: tumor staging; N: limph nodes staging; M: metastasis staging; R: ressection.

A total of 31 patients received postoperative adjuvant chemotherapy, mainly FOLFOX and CAPOX.

During follow-up, 18 patients have died due to GC. The median follow-up was 50 months.

**Figure 1:**
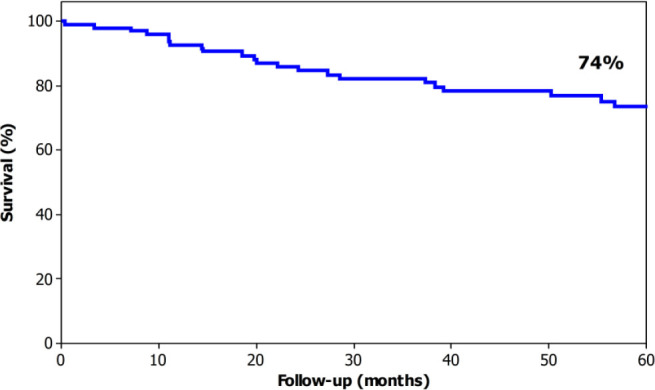
Overall 5-year survival for patients with laparoscopic total gastrectomy for gastric cancer.

**Figure 2:**
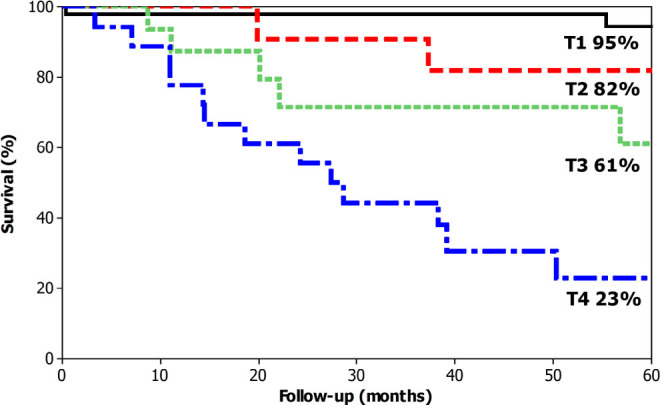
Overall 5-year survival for patients with laparoscopic total gastrectomy for gastric cancer according to the T status.

**Figure 3:**
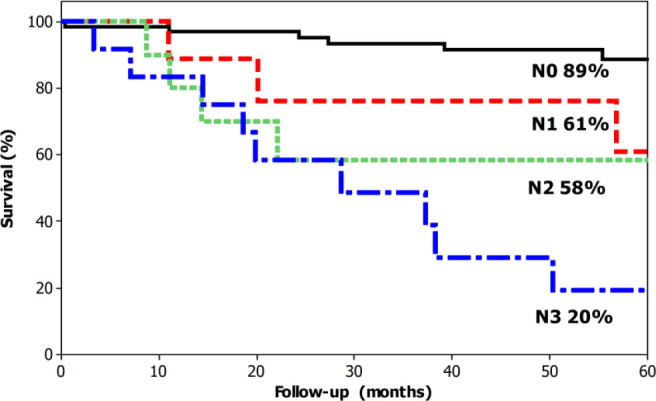
Overall 5-year survival for patients with laparoscopic total gastrectomy for gastric cancer according to the N status

**Figure 4:**
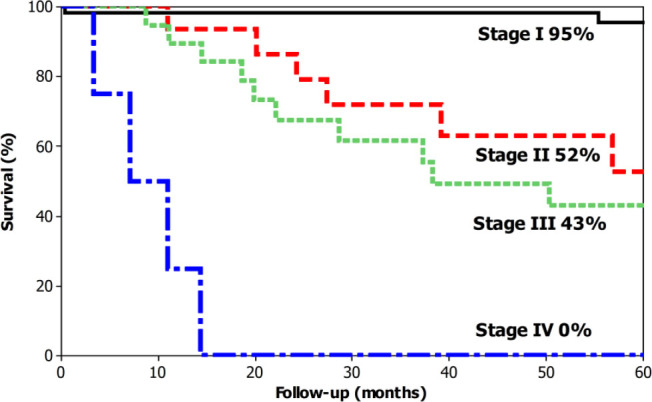
Overall 5-year survival for patients with laparoscopic total gastrectomy for gastric cancer according to the stage.

The overall 5-year survival was 74% (Figure 1). The median survival was 171 months for T1 cases (95% overall 5-year survival), not reached for T2 (82% overall 5-year survival), not reached for T3 (61% overall 5-year survival) and 27 months for T4A (23% overall 5-year survival) (Figure 2). The median survival was not reached for N0 cases (89% overall 5-year survival), 111 months for N1 (61% overall 5-year survival), not reached months for N2 (58% overall 5-year survival), and 28 months (20% overall 5-year survival) (Figure 3). The median survival was not reached for stage I (95% overall 5-year survival), not reached for stage II (52% 5-year overall survival), 38 months for stage III (43% overall 5-year survival), and 7 months for stage IV (0% overall 5-year survival) (Figure 4).

## DISCUSSION

During the past three decades, there has been a great progress in the minimally invasive approach of GC surgery, this development is supported by a large experience from the Eastern countries, mainly Japan^6,17,18,24,25^, Korea^12,13,20,22,38^, and China^11,28,45^. In the West, the development of laparoscopic gastrectomy has been slower, mainly due to a lower global incidence of GC, a lower diagnosis in early stages of GC, more proximally located tumors requiring total gastrectomy, and higher BMI among other factors^41,42^. Thus, this surgical approach has been developed in fewer Western centers^37,41,42^.

In this study, we describe our results with 100 LTG, using a completely laparoscopic approach. There are few reports with this volume of cases as a single center in Western countries^8^. In fact, in Latin America, there are only a few series describing this surgical approach with limited experience^2,15,36,47^. In this series, we present very positive results, with a low conversion rate at 6%, low bleeding at 150 cc, and an operative time of 330 min. The surgical morbidity was lower, compared to prospective series^12,16,42^. And the length of hospital stay of 8 days was similar to the prospective stomach trial from Europe^42^.

This experience has been developed in a high-incidence Western country in a public hospital with a large case volume of GC surgery. The annual number of gastrectomies at our hospital for GC is 60–70 cases per year^34^. The staff surgeons have a large previous experience in open GC surgery and a large experience in benign foregut laparoscopic surgery for hiatal hernia, reflux, and bariatric surgery, which may help in the learning curve of LTG, which represents a difficult surgery, that involves a complex lymph node dissection and a complex reconstruction of the digestive tract, especially in the totally laparoscopic approach.

Compared to our previous historical cohort, with over 1000 gastrectomies, even though it is hard to compare different series, our previous paper described 31% total morbidity^34^, and in this case series, this compared favorably at 26%. And our historical 30-day mortality was 4.6%^34^; also in this series, there is a favorable comparison with only 1% postoperative mortality. This may be due to the surgical approach; however, other factors such as patient selection, surgical experience, and the time period of both studies cannot be ruled out.

When we compare our data to prospective studies like the JCOG1401^16^, which included 195 laparoscopic-assisted total gastrectomies, the operative time was 309 min and major morbidity was 29%, and in our study, operative time was similar and major (grade 3 or higher) morbidity was only 7%, comparing favorably. The prospective KLASS-03^12^ study included 170 patients with an LTG, reported postoperative morbidity of 20% and postoperative mortality at 0.6%, with 9% of patients developing grade 3 or higher morbidity, which is comparable to our study. Both of these prospective studies^12,16^ included only patients in clinical stage I, and a lower BMI at 22 and 24, respectively, unlike our study which included 50% of patients in stage II or higher and a median BMI of 26.

One of the critical steps in LTG is esophago-jejunal anastomosis, and the lack of universally accepted methods is probably one of the major issues in the acceptance of LTG as routine practice^14,21,26,33^. Probably, the most employed method for esophago-jejunal anastomosis is the linear stapler overlap method^12,14^, and one of its main advantages is the higher reproducibility rate, but it has several pitfalls—mainly the need to perform a higher anastomosis in the mediastinal esophagus and the difficulties in the manipulation of the stapler in a narrow space at the level of the hiatus.

There are currently many options for esophago-jejunal anastomosis method^14,21,26,33^, but none of them have proven superiority, and there are no randomized controlled trials comparing the different options and only few comparative studies^3,4,9,10,43,44,46^.

In this study, we used a hand-sewn anastomosis in 90% of cases. We have previously reported our results with this technique, with a leak rate of 3.8%^33^. We developed this anastomosis from a laparoscopic Roux-en-Y gastric bypass performed in our center. In this study, the number of cases with esophago-jejunal anastomosis using a mechanical stapler was only 10, which does not allow a significant comparison of both methods. However, the leak rate was 4% when we used our standard hand-sewn method of esophago-jejunal anastomosis, the same as our initial results^33^. In a recent meta-analysis, there was no difference in leak and stenosis rate when comparing hand-sewn vs. stapled anastomosis after total gastrectomy^10^. However, as described by other groups, we believe that the hand-sewn anastomosis is the most adequate method after LTG, as it allows an intraabdominal anastomosis and it has a low leak and stenosis rate, lower anastomosis time, smaller incisions, and lower hospitalization costs^4,9,43,44^. One disadvantage is that it requires a high experience in intracorporeal laparoscopic suturing and knotting. In the near future with the wider use of barbed sutures^7,44^ and the robotic platform^29,35^, we may see an increase in the adoption of this esophago-jejunal anastomosis method. In the future, there is a need to compare the different esophago-jejunal anastomosis methods in a randomized prospective study to identify the best method.

The oncologic results in our study are in accordance with oncologic principles. The median lymph node count was 38 nodes, which supports the adequate lymph node dissection D1+/D2 performed in most cases. This is comparable to other LTG studies, like 41 lymph nodes resected in the LTG study from Japan JCOG 1401^16^, 35 lymph nodes in the laparoscopic group of the CLASS 02 study^28^, and 41 lymph nodes in the minimally invasive group of the STOMACH study^42^.

In the current study, we performed a long-term follow-up of our patients, with a median follow-up of 50 months. For stage I cases, the survival was 95%, which is very comparable to the series with stage I GC, with survival over 90% at 5 years^17,20^. In more advanced stages II and III, even though there are fewer cases, 17 and 20, respectively, the survival was adequate, at 52 and 43%, respectively. Currently, there is evidence from randomized controlled trials on the oncologic equivalence for clinical advanced GC treated with distal laparoscopic gastrectomy compared to open surgery^6,11,38^, and total gastrectomy has not been validated in an RCT with long-term follow-up. The long-term results of the STOMACH study, which compares LTG to open surgery for advanced gastric cancer after neoadjuvant treatment, are awaited^42^, and currently, the KLASS-06 is an ongoing phase-III randomized trial comparing open and LTG for advanced gastric cancer, results will be available in the next few years. However, our current results are promising for cases in stage II-III. Amongthe possible oncologic benefits of laparoscopic surgery is the lower morbidity rate and faster recovery that would allow an earlier initiation of adjuvant chemotherapy^19,39^.

## CONCLUSIONS

These results support the feasibility and oncological adequacy of minimally invasive total gastrectomy. Postoperative morbidity has an acceptable rate. Long-term survival was in accordance with the disease stage.
